# 

**Published:** 1829-09

**Authors:** 


### MONTHLY LIST OF MEDICAL BOOKS.

An Essay on the Deaf and Dumb, showing the Necessity of Medical Treatment in early Infancy; with Observations on Congenital Deafness. By J. H. Curtis, Esq. &c.—8vo. pp. 211. Longman, London, 1829.

An Introduction to Systematical and Physiological Botany. Illustrated with explanatory Engravings. By THOMAS CASTLE, F.L.S. Surgeon.—12mo. pp. 284. Cox, London, 1829.

The intention of the present work is to give the student a comprehensive outline of the different branches of botanical seience. This purpose Mr. Castles has very neatly and ably executed. A better introduction to the study of botany cannot be consulted.

Medical Botany, No. XXXII. for August 1829. By JOHN STEPHENSON, M.D. F.L.S. &c. and J. M. CHURCHILL, F.L.S. &c.—Published by Tilt, Fleet street.

In this Number of this highly esteemed work, there are excellent engravings and practical descriptions of the qualities, medical properties, uses, and officinal preparations, of the Laurus Sassafras, the Laurus Cinnamomum, the Centaurea Benedicta, and the Pistacia Terebinthus.

Pathological Observations. Part II. On Continued Fever, Ague, Tic Doloureux, Measles, Smallpox, and Dropsy; illustrated by Cases. With a preliminary Dissertation on the Institutes of Medicine, and an Appendix on the Origin, Prevention, and Cure of Organic Diseases. By WM. STOKER, M.D. &c.—8vo. pp. 267. Dublin, 1829.

### NOTICES.

*Communications have been received from* Mr. PEREIRA, Mr. CHENEVIX, &c.

